# Evaluation of B-type Natriuretic Peptide for validation of a heart failure register in primary care

**DOI:** 10.1186/1471-2261-7-23

**Published:** 2007-07-30

**Authors:** Gurbir S Bhatia, Michael D Sosin, Jane Stubley, Jeetesh V Patel, Elizabeth A Hughes, Rebecca Gibbs, Russell C Davis

**Affiliations:** 1 Department of Cardiology, Sandwell & West Birmingham Hospitals NHS Trust, Lyndon, West Bromwich, B71 4HJ, UK; 2 Department of Pathology, Sandwell & West Birmingham Hospitals NHS Trust, Lyndon, West Bromwich, B71 4HJ, UK; 3 Regis Medical Centre, Darby Street, Rowley Regis, West Midlands, B65 0BA, UK

## Abstract

**Background:**

Diagnosing heart failure and left ventricular systolic dysfunction is difficult on clinical grounds alone. We sought to determine the accuracy of a heart failure register in a single primary care practice, and to examine the usefulness of b-type (or brain) natriuretic peptide (BNP) assay for this purpose.

**Methods:**

A register validation audit in a single general practice in the UK was carried out. Of 217 patients on the heart failure register, 56 of 61 patients who had not been previously investigated underwent 12-lead electrocardiography and echocardiography within the practice site. Plasma was obtained for BNP assay from 45 subjects, and its performance in identifying echocardiographic abnormalities consistent with heart failure was assessed by analysing area under receiver operator characteristic (ROC) curves.

**Results:**

30/217 were found to have no evidence to suggest heart failure on notes review and were probably incorrectly coded. 70/112 who were previously investigated were confirmed to have heart failure. Of those not previously investigated, 24/56 (42.9%) who attended for the study had echocardiographic left ventricular systolic dysfunction. A further 8 (14.3%) had normal systolic function, but had left ventricular hypertrophy or significant valve disease. Overall, echocardiographic features consistent with heart failure were found in only 102/203 (50.2%). BNP was poor at discriminating those with and without systolic dysfunction (area under ROC curve 0.612), and those with and without any significant echocardiographic abnormality (area under ROC curve 0.723).

**Conclusion:**

In this practice, half of the registered patients did not have significant cardiac dysfunction. On-site echocardiography identifies patients who can be removed from the heart failure register. The use of BNP assay to determine which patients require echocardiography is not supported by these data.

## Background

Heart failure is an increasingly prevalent condition, associated with markedly reduced survival and quality of life despite recent advances in management [1]. The prevalence of heart failure in the community is around 2%, and a similar proportion has left ventricular systolic dysfunction (LVSD, an important precursor of heart failure) [2]. In 1996, estimated overall incidence rate for heart failure in the general population was 4.4 per 1000 person-years in men and 3.9 per 1000 person-years in women [3].

The associated frequent hospitalisations and long-term, evidence-based pharmacological therapies render heart failure a costly condition to treat: recently, heart failure was estimated to account for almost 2% of total NHS expenditure [4]. Given the costs, therefore, it is vital that such patients are correctly identified. Unfortunately, it is well recognised that diagnosing heart failure and LVSD upon clinical grounds alone is beset with difficulties due to the limited specificity and sensitivity of typical symptoms. Modern diagnostic criteria for heart failure stipulate the need to identify objective evidence of cardiac dysfunction [5]. Indeed, in the United Kingdom, the National Institute for Clinical Excellence (NICE) has recommended confirmation of the presence of cardiac dysfunction using echocardiography [6]. Moreover, planned service contractual changes should further encourage general practitioners to request such investigations.

Within primary care practices, the compilation of registers recording the details of patients with a variety of chronic diseases (including heart failure) is common. There is likely to be much heterogeneity among patients placed upon such a register. For example, patients discharged from hospital are more likely to have had diagnostic investigations, whereas community subjects, in whom access to echocardiography might be limited, may have been registered purely on the presence of symptoms or their perceived improvement with diuretic treatment; high false-positive rates for diagnosis of heart failure are apparent in the latter group [7,8]. A further key recommendation of the NICE guidelines for heart failure is that historical diagnoses should be reviewed, and subsequently, only confirmed cases should receive recommended treatment [6].

With limited community access to echocardiography, and high volumes of requests for echocardiography within hospitals, more practical (and cheaper) methods of identifying cardiac dysfunction are clearly attractive. Thus, the usefulness of quantifying plasma levels of natriuretic peptides has received much attention in recent years. These markers are thought to be released from myocardium in response to wall stretch, with levels correlating with severity of heart failure. Brain natriuretic peptide (BNP) measurement is a valuable adjunct to clinical assessment in distinguishing heart failure from non-cardiac causes of acute dyspnoea in patients presenting to emergency care [9,10]. However, its role in primary care is less clear [11]. Its use in exclusion of heart failure is advocated in the NICE guidelines [6].

We aimed to assess the accuracy of diagnosis among a sample of patients registered as having heart failure in a single primary care practice. The feasibility of performing portable echocardiography within the primary care site and the potential value of BNP assay in this group were also studied.

## Methods

A register of all practice patients with a previous diagnosis coded as heart failure (Read code G58) was compiled in November 2003. A search of practice and hospital records was done to identify patients who had already undergone objective assessment of cardiac function by echocardiography or other investigation. Patients who had not previously had any such investigation were invited to attend for electrocardiography, venepuncture, and echocardiography, all performed in one visit to the general practice.

Demographic data and previous medical history were recorded by nursing staff, and current medications were documented from current prescription records. Standard 12-lead ECG and blood pressure measurement was performed by nursing staff. The presence of major (pathological Q-waves, left bundle branch block, LVH, atrial fibrillation) and minor abnormalities (all other deviations from normal) were recorded by two investigators (GSB or MDS). Subjects underwent trans-thoracic echocardiography performed by one of two investigators (GSB or MDS) using portable equipment (Acuson Cypress, Siemens). Left ventricular systolic function was analysed qualitatively based upon standard parasternal and apical views, with left ventricular systolic dysfunction defined as left ventricular ejection fraction < 40%. Valvular regurgitation was assessed semi-quantitatively based upon colour and continuous wave Doppler flow characteristics. Regurgitant lesions of at least moderate severity were considered significant. Mitral and aortic stenosis were significant if mitral valve area was < 1.5 cm^2 ^or aortic valve gradient was > 20 mmHg, respectively. Echocardiograms were reported by GSB and MDS, consistent with the local hospital-based clinical echocardiography service.

Following echocardiography, with patients still supine and resting, non-fasting venous blood was taken into EDTA tubes and refrigerated (4°C) overnight. The tubes were transported to the local hospital laboratory for centrifugation. Plasma was subsequently frozen at -80°C prior to BNP immunochemiluminometric assay (ADVIA Centaur autoanalyser, Bayer Diagnostics). The intra-assay coefficient of variation was 6.1% at 12.6 pmol/L, 3.2% at 118.0 pmol/L, and 4.0% at 474.5 pmol/L.

Ethical approval was obtained by the Sandwell Local Ethics Committee.

### Statistical analysis

Comparisons of normally and non-normally distributed data were made using the Students t-test and Mann-Whitney test, respectively. Categorical data were compared using the χ^2 ^test. For assessment of diagnostic performance of the BNP assay, receiver operator characteristic (ROC) curves were constructed by plotting sensitivity versus 1-specificity. A cut-off that provided the best combination of sensitivity and specificity was ascertained, and negative (NPV) and positive (PPV) predictive values were calculated for this level. P values < 0.05 were deemed to indicate statistical significance. Data were analysed using SPSS version 10.0 for Windows.

## Results

**Figure 1:**
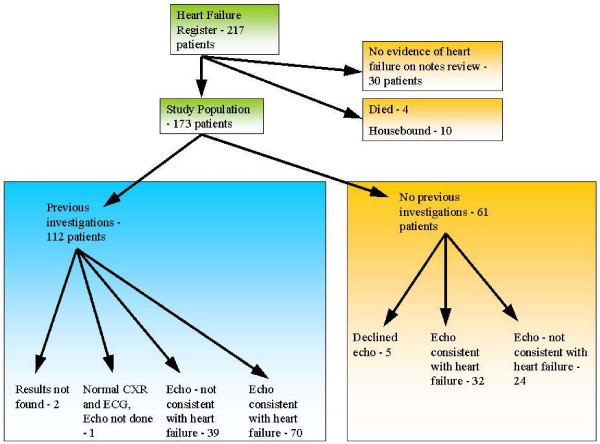
Flow diagram for the 217 patients from the heart failure register.

217 patients were registered with a previous diagnosis of heart failure (see Figure 1). Upon careful review of practice records, 30 patients did not have any symptomatic or other evidence supporting a diagnosis of heart failure, and these patients were removed from the register. Ten patients were considered too unfit or unwell to attend the practice, whilst a further four patients died between compilation of the register and the next step of the study. Of the remaining 173 patients, 112 had a record of previous heart failure investigations. 70 of these patients had echocardiography reports consistent with heart failure whereas echocardiography in 39 patients had not supported the diagnosis of heart failure. One patient was considered unlikely to have heart failure due to a documented normal ECG and chest X-ray. We were unable to trace results of two patients. Of the remaining 61, 56 agreed to attend for further investigations.

The mean age of the 56 patients attending for echocardiography was 77 years (range 54–99), and 30/56 (53.6%) were female. Baseline characteristics of the patients are presented in Table 1. 24 (42.9%) were found to have LVSD. A further eight (14.3%) had normal systolic function, but had an additional echocardiographic diagnosis (left ventricular hypertrophy, or significant valve disease), consistent with a diagnosis of heart failure with preserved systolic function. Isolated diastolic dysfunction, in the absence of systolic dysfunction, valve disease or left ventricular hypertrophy, was not specifically examined for.

**Table 1:** Baseline characteristics of patients with and without echocardiographic abnormality indicative of heart failure

	Echocardiogram not consistent with heart failure (n = 24)	Echocardiogram consistent with heart failure (n = 32)	p value
Age – mean (SD)	75.4 (7.2)	78.3 (8.4)	0.184 (t-test)
			
Male gender	13 (54.2%)	13 (40.6%)	0.315
Ischaemic heart disease	7 (29.2%)	16 (50.0%)	0.117
Hypertension	12 (50.0%)	14 (43.8%)	0.643
Diabetes	4 (16.7%)	9 (28.1%)	0.315
Atrial fibrillation	3 (12.5%)	6 (18.8%)	0.529
Peripheral vascular disease	2 (8.3%)	2 (6.3%)	0.765
Chronic airflow limitation	6 (25.0%)	5 (15.6%)	0.382
Diuretic	21 (87.5%)	24 (75.0%)	0.244
Angiotensin converting enzyme inhibitor or Angiotensin receptor blocker	14 (58.3%)	23 (71.9%)	0.290
Beta-blocker	2 (8.3%)	8 (25.0%)	0.107
Statin	9 (37.5%)	14 (43.8%)	0.638
Abnormal ECG	13 (54.2%)	27 (84.4%)	0.013
BNP (pmol/l) median (interquartile range)	11.7 (20.0)	32.75 (35.8)	0.010 (Mann-Whitney test)

Note – values are number (percentage) unless stated. P values refer to chi-squared tests unless stated.

The groups were similar in age and gender. Patients with echocardiography consistent with heart failure appeared more likely to have a past medical history of ischaemic heart disease, although this was not statistically significant. Of note, only 5 patients with echocardiography consistent with heart failure had a normal ECG, whereas 11 patients in whom heart failure was excluded had a normal ECG. Therefore the positive predictive value of an abnormal ECG was 0.68, whilst the negative predictive value was 0.69.

Overall, objective evidence for a diagnosis of heart failure was confirmed in only 102 (50.2%) of those on the practice register (excluding the 14 who died or were too unwell to take part).

**Figure 2:**
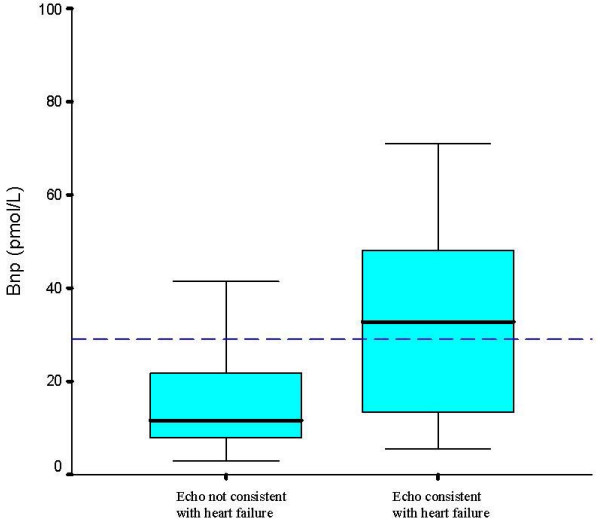
**Distribution of BNP results in patients with and without echocardiographic abnormality indicative of heart failure**. Reference line at 29pmol/l indicates cutoff below which heart failure is ruled out in patients with acute shortness of breath in previous studies.[9]

**Figure 3:**
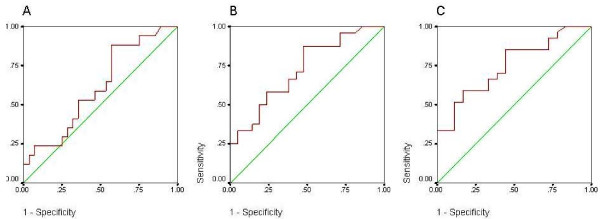
**Receiver operating characteristic curves for BNP**. A. Discrimination of those with and without left ventricular systolic dysfunction B. Discrimination of those with and without left ventricular systolic dysfunction ***or ***other significant echocardiographic abnormality C. Discrimination of those with and without left ventricular systolic dysfunction ***or ***other significant echocardiographic abnormality ***or ***atrial fibrillation.

Blood sampling was performed in 45 patients who attended for echocardiography. BNP was significantly higher in the patients with echocardiographic features consistent with heart failure, although in both groups the median BNP was lower than that seen in acute heart failure, and even in those with echocardiographic features consistent with heart failure the median BNP was similar to the cut-off proposed for ruling out heart failure in acutely breathless patients or untreated clinic patients (29pmol/l) [12]. The distribution of BNP results for those with and without echocardiographic abnormality is shown in Figure 2. ROC curve analysis showed BNP to be a poor discriminator between those with and without left ventricular systolic dysfunction (area under the curve 0.612, Figure 3A). A larger area under the curve was found when left ventricular systolic dysfunction and other echocardiographic abnormalities (significant valve disease or left ventricular hypertrophy) were considered (area 0.723, Figure 3B), and when those with atrial fibrillation (who ideally require echocardiographic examination anyway [13]) who had not already been included were added in to the model (area 0.750, Figure 3C).

## Discussion

### Summary of main findings

On a first 'trawl' of practice records, there appeared to be several (30, 13.8%) patients who were wrongly placed upon the heart failure register. For example, these patients may have complained of mild peripheral oedema alone or dyspnoea responsive to bronchodilators. Initially, it appeared that many patients had never undergone echocardiography or other diagnostic imaging. However, over half (112/203) were found to have undergone echocardiography when all patients' correspondence and hospital databases had been searched. Most such patients will not need to have repeated echocardiography performed if registers are to be validated, so a comprehensive record search, although labour-intensive, is likely to be very useful. Notably, 40 patients remained on the register despite documented investigations showing no cardiac dysfunction.

We were able to identify 61 patients labelled with heart failure but with no record of objective investigations to confirm or refute the diagnosis. Many of these patients were taking heart failure drugs, although the evidence base for many of these drugs in heart failure is only for those with left ventricular systolic impairment. Drugs such as vasodilators and diuretics rapidly lower BNP in patients with acute heart failure [14], and angiotensin converting enzyme inhibitors [15] and beta-blockers [16] lower BNP in patients with chronic heart failure, and so the difference in BNP between patients with and without left ventricular systolic dysfunction in our study might have been greater if tested prior to commencing anti-failure medication. Ideally, all patients with a label of heart failure would undergo echocardiography to identify those with left ventricular systolic dysfunction, or significant valvulopathy. However, echocardiography is time consuming and requires expensive equipment and highly trained operators. BNP is attractive as a method of selecting a subgroup of patients who require echocardiography, but our data do not support the use of this test in this setting.

### Comparison with existing literature

One previous study of 100 stable patients with a label of heart failure in a general practice setting also concluded that BNP was of limited value in the diagnosis of left ventricular systolic dysfunction in this situation [11]. A second study, using N-terminal pro-BNP rather than BNP reported a similar area under the receiver operating characteristic curve (0.8) for the identification of left ventricular systolic dysfunction in a general practice population with a label of heart failure (n = 103) [17]. Natriuretic peptides may be useful in assisting diagnosis of patients with symptoms of heart failure at first presentation to their general practitioner, before they are commenced on treatments that might lower BNP [18,19]. Further studies would be necessary to confirm this possibility.

### Strengths and the limitations of this study

Performing portable echocardiography within a primary care site was found to be feasible, and particularly appealing to many of the elderly patients who lived considerable distances from secondary care centres. Furthermore, blood testing was undertaken at the practice, with samples refrigerated overnight prior to transportation for centrifugation and freezing prior to batch testing. It is possible that there may be some deterioration in BNP levels with such an approach, however our intention was to replicate the likely situation should BNP assays become a routine primary care test. Had we centrifuged and frozen the sample immediately, the performance of BNP might have been enhanced, but the results would not then have been applicable to the 'real world'. A similar approach to that used in our study was used in a multicentre study of BNP levels in patients with heart failure [20]. We recognise that this study was of limited size, being performed in a single, albeit large, general practice. Detailed study of left ventricular diastolic function was not done as part of the echocardiograms. However, left ventricular hypertrophy, a common cause of diastolic heart failure, is reported on. We felt it most appropriate to concentrate on left ventricular systolic dysfunction, to which the NICE guideline [6] and most of the evidence base in heart failure applies.

### Implications for clinical practice

We cannot support the use of BNP testing in validation of general practice heart failure registers. If access to BNP testing for general practitioners – as advocated in the NICE heart failure guidelines – becomes widespread, it is important to appreciate that the test is not likely to be useful in patients with a label of heart failure who are already on treatment. A normal ECG, however, retains its negative predictive value for systolic dysfunction.

## Conclusion

Heart failure is difficult to diagnose and NICE guidelines advocate confirmation of diagnoses made previously. Only half of those patients registered as having heart failure in a primary care practice had the diagnosis validated by on-site echocardiography. Plasma BNP assay did not appear to be a useful tool for retrospective diagnostic validation in patients already on treatment for heart failure.

## Competing interests

All authors have attended medical meetings sponsored by various pharmaceutical companies. GSB and MDS have received honoraria for speaking at meetings hosted by Bayer Diagnostics.

## Authors' contributions

RG had the original idea for the study, compiled the register, and carried out searches of practice notes. RCD, JS, MDS, and GSB developed the study protocol. JS, RG, MDS, GSB carried out the search for results of previous investigations. JKP and EAH, assisted by GSB and MDS, carried out BNP assays. GSB and MDS carried out echocardiography and data analysis and wrote the paper. All authors contributed to the final version of the paper. RCD will act as guarantor.

## Pre-publication history

The pre-publication history for this paper can be accessed here:


